# Genome evolution of Mediterranean olives following DNA repeats diversification

**DOI:** 10.1002/tpg2.70323

**Published:** 2026-10-15

**Authors:** Flavia Mascagni, Samuel Simoni, Gabriele Usai, Alexis Ryo, Marco Castellacci, Ugo Rogo, Marilena Ceccarelli, Luciana Baldoni, Tommaso Giordani, Lucia Natali, Andrea Cavallini, Guillaume Besnard

**Affiliations:** ^1^ Department of Agriculture, Food and Environment University of Pisa Pisa Italy; ^2^ Centre de Recherche sur la Biodiversité et l'Environnement (CRBE) Université de Toulouse, CNRS, IRD, Toulouse INP, Université Toulouse 3–Paul Sabatier (UT3) Toulouse France; ^3^ Department of Chemistry, Biology and Biotechnology University of Perugia Perugia Italy

## Abstract

The olive tree (*Olea europaea* L. subsp. *europaea*) is one of the most representative perennial crops of the Mediterranean Basin, yet the genomic processes underlying its domestication and diversification remain poorly understood. Here, we investigated repeatome variation across 20 accessions, including 11 wild oleasters and nine cultivated varieties, using comparative clustering of high‐throughput sequencing data, complemented by structural analyses of transposable elements (TEs) and tandem repeats (TRs). Repetitive DNA accounts for about 54% of the olive genomes and is composed primarily of long terminal repeat retrotransposons (LTR‐REs) and TRs. Comparative analyses revealed lineage‐specific differences in LTR‐REs between wild and cultivated genotypes, while TRs exhibited higher abundances and greater variability in oleasters for some families (e.g., Oe‐80 and Oe‐178), reinforcing the view that TRs evolve rapidly and largely independently in olive. We also identified a new TR (Oe‐155) and confirmed its distinct monomer sequence and chromosomal organization, suggesting that this subfamily is differentiating from TRs Oe‐218 and Oe‐80. To refine TE dynamics, we surveyed full‐length elements in two reference assemblies (cv. Leccino and a wild *sylvestris* genotype). Leccino is enriched in intact LTR‐REs, whereas *sylvestris* harbored more full‐length Class II DNA transposons. Additionally, the patterns of TE‐gene proximity vary significantly between them. Dating of full‐length LTR‐RE insertions revealed that most RE activity predates domestication, with only a small fraction of very recent insertions. These results highlight repetitive DNA as a major contributor to genome diversification in olive and provide a foundation for integrating repeatome variation with demographic history, introgression, and clonal propagation.

## Plain Language Summary

The olive tree is a key crop in the Mediterranean region, but the genetic processes that shaped its diversity and domestication remain poorly understood. In this study, we compared the genomes of wild and cultivated olives to investigate the role of repetitive DNA. We found that more than half of the olive genome consists of repetitive sequences, mainly transposable elements, and tandem repeats. These components differ between wild and cultivated olives: some transposable elements show lineage‐specific patterns, while certain tandem repeat families are more abundant and variable in wild populations. We also identified a new tandem repeat subfamily, localized by fluorescence in situ hybridization, and found that most transposable element activity occurred before domestication. Overall, our results show that repetitive DNA plays a major role in shaping the olive genome structure and diversity, providing a basis for future studies of olive evolution and genetic variation.

**Abbreviations**
DAPI: 4′,6‐diamidino‐2‐phenylindole
FISH: fluorescence in situ hybridization
KEGG: Kyoto encyclopedia of genes and genomes
LTR: long terminal repeat
MYA: million years ago
RE: retrotransposon
SSC: saline‐sodium citrate
SSR: simple sequence repeat
TE: transposable element
TR: tandem repeat

## 1 INTRODUCTION

The Mediterranean olive tree (*Olea europaea* L. subsp. *europaea*; Oleaceae) is a long‐lived perennial species of agricultural, ecological, and cultural significance in the Mediterranean basin, serving as a primary source of olive oil and contributing to the cultural heritage of the area (Kaniewski et al., 2012; Loumou & Giourga, 2003; Zohary et al., 2012). Taxonomically, two varieties of *O. e. europaea* are recognized (Green, 2002): (i) the cultivated form (*O. e. europaea* var. *europaea*), mainly propagated vegetatively, and (ii) the spontaneous form (*O. e. europaea* var. *sylvestris* (Mill.) Lehr.) that reproduces sexually. The latter, commonly called oleaster, is widespread throughout the Mediterranean and corresponds to both genuinely wild and feral olives (Besnard et al., 2013; Diez et al., 2015; Green, 2002).

Because of centuries of cultivation and human selection, the olive domestication history remains the subject of ongoing debate. Genomic evidence suggests complex scenarios involving recurrent admixture, introgression, and possibly multiple domestication events. While traditional hypotheses proposed a single domestication center in the eastern Mediterranean, population genomic studies have pointed toward a reticulate domestication process in which cultivated olives have continuously interbred with wild populations across different regions of the Mediterranean, which has blurred the genomic distinction between wild and cultivated forms, complicating the identification of clear domestication signatures (Besnard et al., 2001, 2013; Diez et al., 2015; Gros‐Balthazard et al., 2019; Julca et al., 2020; Mariotti et al., 2023; Rubio de Casas et al., 2006).

In this context, the structural features of the genome, such as repetitive DNA elements, may provide additional insights to clarify aspects of *Olea* evolution and domestication. As is well known, a very large portion of most eukaryotic genomes is composed of repetitive DNA sequences, which can be organized either as interspersed repeats or in tandem arrays. The amplification or loss of these sequences contributes to the remarkable variation in genome size and structure, even among closely related species within the same genus (Ammiraju et al., 2007; Hendrix & Stewart, 2005; Mascagni et al., 2015; Price et al., 2005).

Transposable elements (TEs), or transposons, are mobile DNA sequences that can change their chromosomal location, with the potential of affecting the host genome either during mobilization events or after their insertion. TEs are classified into two classes, according to whether their transposition intermediate is RNA (Class I or retrotransposons [REs]) or DNA (Class II or DNA TEs; Wicker et al., 2007). Despite differences in transposition mechanisms and genomic abundance, REs and DNA TEs can introduce genetic variation, acting as an endogenous driver of evolvability that would otherwise be unattainable (Lisch, 2013). TEs are responsible for rapid genome remodeling by creating chromosome rearrangements and new regulatory gene networks (Lisch, 2013), thereby initiating reproductive isolation. The mutagenic action of transposons carries out substantial genetic variability (Morgante et al., 2007), novel functions by fine‐tuning gene activity (Morgante et al., 2005; Slotkin & Martienssen, 2007), and, aside from polyploidy, TEs are the major drivers of genome size variation (Hawkins et al., 2006; Neumann et al., 2006; Piegu et al., 2006). Moreover, TEs can also be exapted and give rise to novel functional genes (Joly‐Lopez & Bureau, 2018; Ventimiglia et al., 2023). In plants, REs are the most prevalent repeats, representing a substantial portion of the genomic content (Lisch, 2013; Vitte et al., 2014). The most abundant RE group belongs to long‐terminal repeats (or LTR‐REs), which fall into two main superfamilies, *Gypsy* and *Copia*, distinguished by the arrangement of protein domains within their encoded polyproteins (Wicker et al., 2007). These superfamilies can be further classified into distinct evolutionary lineages, with *Copia* comprising seven major lineages, and *Gypsy* including three principal ones (Llorens et al., 2010; Mascagni et al., 2020; Neumann et al., 2019; Vangelisti et al., 2019; Wicker & Keller, 2007).

Compared to interspersed genome repeats, other types of repetitive sequences generally represent a smaller fraction of plant genomes and usually play a more limited role in shaping genome size and architecture. Among these, tandem repeats (TRs) are sequences composed of adjacent, repeating units that often differ in guanine and cytosine content from the surrounding genomic DNA (Szybalski, 1968). Initially identified through satellite bands in gradient centrifugation experiments, these sequences are commonly referred to as satellite DNA (T. Schmidt & Heslop‐Harrison, 1998). These sequences are predominantly found in heterochromatic regions, where they contribute to the formation of essential chromosomal structures such as centromeres and telomeres (Garrido‐Ramos, 2017; Hartley & O'Neill, 2019). They are also enriched in pericentromeric regions and at chromosomal breakpoints, where they may promote genome instability and contribute to structural rearrangements, including inversions and recombination events (Flores et al., 2007; Hirabayashi & Owens, 2023). They can also contribute to genetic disorders by affecting gene regulation (Horton et al., 2023). TR families are highly variable in terms of their repeat unit length, genomic distribution, and abundance, reflecting a high rate of evolutionary turnover, which is also due to their propensity to undergo changes in copy number resulting from polymerase slippage during replication or unequal recombination (Melters et al., 2013; Thakur et al., 2021). As a result, most satellite DNA families tend to be species‐ or genus‐specific (Garrido‐Ramos, 2015), and this feature has thus been used to trace back the origin of genomic blocks in early‐generation crop hybrids, even enabling the identification of an unknown progenitor in the Australasian sugarcane (Garsmeur et al., 2025). However, exceptions also exist with long‐term sequence conservation of some satellite families across distantly related taxa (del Bosque et al., 2013, 2014; Cafasso & Chinali, 2014; Mehrotra et al., 2014), suggesting conserved functional roles in genome organization and regulation (Pezer et al., 2012; Plohl et al., 2012).

In the cultivated olive (*O. e. europaea* var. *europaea*), current evidence supports a unique pattern of genome evolution marked by paleo‐polyploidization events and dynamic changes in TE content, alongside a massive proliferation of the TR fraction (Bitonti et al., 1999; Katsiotis et al., 1998). This has resulted in TRs accounting for nearly one‐third of the olive genome (Barghini et al., 2014; Mascagni et al., 2022), a proportion notably higher than in most sequenced plant species, where TRs typically represent less than 10% of the genome, with a few exceptions, such as cucumber (*Cucumis sativus*) and *Fritillaria falcata*, where TRs constitute approximately 23% and 36% of the genome, respectively (Ambrožová et al., 2011; Huang et al., 2009). The released genome assemblies for the cultivated varieties Farga, Leccino, and Frantoio featured a limited characterization of the repeated component (Cruz et al., 2016; Lv et al., 2024; Sebastiani et al., 2025); then, a genome sequence and annotation of a Turkish oleaster (*O. e. europaea* var. *sylvestris*) showed a significantly lower abundance of TRs than expected (Unver et al., 2017). The most recent studies related to the genome of cultivated olive revealed genomic variability resulting from a significant activation of TEs during the domestication process (Jiménez‐Ruiz et al., 2020), but they made only little progress in deciphering the complex structure of its repetitive component (Rao et al., 2021). The difficulty in identifying satellite sequences might be explained by repeat collapse during the genome assembly, leading to the incorrect gauging of the number of repeat copies in a genome, ultimately providing a reference with too few repeat copies (Phillippy et al., 2008).

New possibilities for investigating repetitive sequences in genomes were provided by massive parallel DNA sequencing techniques. Using a computational framework, the identification of the different types of repetitive elements is now possible and allows the study of the dynamics that have changed the repetitive landscape of the *Olea* genome. However, even in studies relying on high‐quality reference genomes assembled using long‐read technologies, a detailed characterization of TRs remains elusive, largely due to the inherent repetitive and arrayed nature of these sequences, which complicates their sequencing, assembly, and annotation. Previous studies have investigated the TR component within the *Olea* genus, leading to the isolation of 11 TR families from genomic libraries of various taxa, with several of them also being cytogenetically mapped using in situ hybridization (Barghini et al., 2014; Bitonti et al., 1999; Contento et al., 2002; Katsiotis et al., 1998; Lorite et al., 2001; Mascagni et al., 2022; Minelli et al., 2000). The high abundance of TRs was also shown to be a general feature of all the analyzed species within subgenus *Olea*, with *O. europaea* subsp. *cuspidata*, now referred to as *O. europaea* subsp. *africana* (Besnard et al., 2024), displaying TR content covering up to 50% of its genome (Mascagni et al., 2022). These findings further supported the hypothesis of a unique evolutionary trajectory within subgenus *Olea*. By contrast, *Fraxinus excelsior*, another member of the Oleeae tribe (subtribe Fraxininae) that diverged from the *Olea* lineage (subtribe Oleinae) in the Eocene (Dupin et al., 2024), shows a genome almost entirely devoid of TRs, suggesting that the massive TR amplification observed in olive likely occurred within the last 40 million years. Interspersed repeats also represent a significant portion of *Olea* genomes, although it is noteworthy that overall, TR abundance was inversely correlated with RE content across the analyzed species (Mascagni et al., 2022). This pattern may indicate a sort of competitive relationship between the two repeat classes, suggesting that species of subgenus *Olea* experienced TR amplification alongside reduced proliferation of REs.

With these unique genomic features, the olive genome may serve as a valuable model for exploring the evolutionary dynamics of plant genome organization. Nevertheless, comparative analyses of the repetitive DNA landscapes between cultivated and wild olives remain limited. Here, we present a study that extends the analysis of repeat variation to include both cultivated varieties and oleasters from the Mediterranean Basin, proposing an investigation into the structure and evolution of olive genomes essential to better understand the mechanisms underlying genome expansion and the functional roles of repetitive elements. Our approach consisted of two steps: (1) an analysis of short‐read sequencing data to analyze the repeatome among cultivated and wild accessions representative of the Mediterranean olive tree diversity, and (2) an analysis of two reference genomes (wild and cultivated), in particular to recover full‐length TEs, analyze their integration over time, and investigate their proximity to genes.

## 2 MATERIALS AND METHODS

### 2.1 Characterization of the repeatomes with short‐read sequencing data

#### 2.1.1 Plant material, DNA sequencing, and preliminary genetic clustering analysis

For this study, leaves from 11 oleasters and nine olive cultivars were collected. Six oleaster accessions (i.e., Al Ascharinah 9, Urla 6, Bni Harchen 1, Dar Chaoui 1, Talambote 2, and Tarifa 1) are maintained in the living olive collection of CEFE Montpellier. Plant material of olive cultivars (leaves and root apices, the latter collected from cuttings) was provided by the Olive Collection of CNR—Institute of Biosciences and Bioresources, Division of Perugia (Perugia, Italy), for DNA sequencing and fluorescence in situ hybridization (FISH) analyses.

Total genomic DNA was extracted from approximately 20 mg of fresh leaves using the BioSprint 15 DNA Plant kit (Qiagen Inc.), according to the manufacturer's instructions. The DNA libraries were obtained using the Illumina TruSeq Nano DNA LT Sample Prep kit according to the manufacturer's protocol (Illumina Inc.), as described in Olofsson et al. (2019). All libraries were sequenced using the Illumina HiSeq 3000 platform, yielding paired‐end reads. Subsequently, FastQC v0.11.9 (https://www.bioinformatics.babraham.ac.uk/projects/fastqc) was used to assess the quality of the sequence reads. Trimmomatic v0.39 (Bolger et al., 2014) was used to remove Illumina adapters and low‐quality reads, and the reads were trimmed at 100 bp in length with the following parameters: ILLUMINACLIP:adapters trimmomatic.fasta:2:30:10 LEADING:3 TRAILING:3 SLIDINGWINDOW:4:15 HEADCROP:10 CROP:100 MINLEN:100.

All reads containing organellar DNA sequences were removed using CLC‐BIO Genomic Workbench 9.5.3 (CLC‐BIO) against a library consisting of chloroplast (GU931818.1, NC_015401.1, and NC_013707.2) and mitochondrial (MG372117.1, MG372118.1, and MG372119.1) sequences of *O. europaea*. Details on all sequenced accessions and the corresponding number of retained reads are reported in Table S1.

To assign each individual to known gene pools in the Mediterranean basin (i.e., eastern vs. western wild gene pools, or cultivated gene pools; Besnard et al., 2018; Diez et al., 2015; Khadari et al., 2019), individuals were preliminarily characterized with 25 microsatellite markers (simple sequence repeats [SSRs]) and included in a genotype database (i.e., 241 genotypes from across the Mediterranean, of which 141 are cultivated and 100 are wild; genotyping methods and SSR dataset are described in Table S2). A clustering analysis was performed using structure v2.3.4 (Pritchard et al., 2000) under the admixture model (mixing of gametes possible at reproduction) for a duration of 100,000 generations after a burn‐in period of 50,000 generations. The analysis was performed for a number of clusters (*K*) ranging from 1 to 10 with 15 iterations for each value of *K*. The most likely number of clusters was determined using the ad hoc measure of Δ*K* performed with structure selector (Evanno et al., 2005; Y. L. Li & Liu, 2018). Each genotype was thus assigned to their respective clusters with a posterior membership coefficient (*p*). When this membership coefficient was superior to 80% for a given cluster and inferior to 10% for any other cluster, the individual was considered properly assigned; otherwise, it was considered admixed between different gene pools. Genetic clusters (see Section 3) were named following Diez et al. (2015), who distinguished three main cultivated groups (i.e., Q1, Q2, and Q3, mainly from the western, central, and eastern Mediterranean, respectively), and two wild groups (i.e., wild western [WW] and wild eastern [WE], from the western and eastern Mediterranean, respectively).

#### 2.1.2 Repeat characterization of cultivated and wild olive accessions using Illumina reads

A sequence similarity‐based clustering method was applied using RepeatExplorer2 (Novák et al., 2020; https://repeatexplorer‐elixir.cerit‐sc.cz/), which enables the de novo identification of repeats. Their proportions were estimated in each genome through a comparative analysis of repetitive elements from the 20 wild and cultivated accessions, with and without satellite filtering. A random set of 1,000,000 sequences was used for each accession. RepeatExplorer2 output was analyzed to collect the clusters identified as repeats. Similarity searches on the remaining unclassified clusters were performed using BLASTN and tBLASTX of the BLAST tool v2.6.0+ (Altschul et al., 1990) against a library of 254 putative full‐length REs of olive to increase the number of annotated clusters (Barghini et al., 2015). Putative satellite repeats (i.e., TRs), identified by RepeatExplorer, were collected for each accession. The monomer sequences of selected satellites were identified with TAREAN (Novák et al., 2017) and manually curated.

#### 2.1.3 Mapping procedure for estimating genome proportion of repeated elements

The genome proportion of each type of repeated element was measured by calculating their abundance values. The estimation was carried out by counting the number of reads mapping into clusters of TEs and the library of TR sequences. The TR library was cleaned of redundant sequences using CD‐HIT v4.7 (W. Li & Godzik, 2006) with a 90% identity threshold. The mapped reads count was normalized per million total reads by dividing the mapped reads by the total number of trimmed reads in each library. CLC‐BIO Genomic Workbench was used to map the reads of each library with the following parameters: mismatch cost = 1, deletion cost = 1, insertion cost = 1, similarity = 0.7, and length fraction = 0.7. This method had already been used for many plant species (Mascagni et al., 2015; Mascagni, Usai, et al., 2018; Natali et al., 2013; Tenaillon et al., 2011), including olive (Barghini et al., 2014, 2015; Mascagni et al., 2022). All statistical analyses were performed in R (v4.3.2) using the “stats” package. Statistical significance was set at *p* < 0.05.

#### 2.1.4 Phylogenetic analysis of TRs

A phylogenetic tree was constructed using TR sequences from the 20 genotypes (adding those from the *Olea* TR dataset published in Mascagni et al. [2022]), based on a multiple sequence alignment performed with Clustal X v2.1 (Larkin et al., 2007) and using the neighbor‐joining clustering method with 1000 bootstrap replications. The resulting tree was visualized using FigTree v1.4.4 (Rambaut, 2018).

#### 2.1.5 Fluorescence in situ hybridization

FISH experiments were carried out to analyze the genomic distribution of the Oe‐155 element and its closely related Oe‐218 (for comparison). The Oe‐155 probe (Table S3), designed on the specific Oe‐155 TR of *O. europaea* and consisting of an arranged array of four monomer repetitions, was synthesized and cloned into the pUC57 vector by Synbio Technologies. Oe‐155 or Oe‐218 probes (1 µg each) were labeled by nick translation using either DIG‐Nick Translation Mix (Roche) or Biotin‐Nick Translation Mix (Roche). Root apices from cuttings of cv. Leccino were treated with a saturated aqueous solution of alpha‐bromonaphthalene for 4 h at room temperature and fixed in ethanol‐acetic acid (3:1 v/v). Slides were prepared using root apices washed in an aqueous solution of 6 mM sodium citrate and 4 mM citric acid, treated with a mixture of 10% pectinase (Sigma), 2% macerozyme (Serva), and 10% cellulase (Calbiochem) in citrate buffer pH 4.6 for 2 h at 37°C, and then squashed under a coverslip in a drop of 60% acetic acid. The coverslips were removed after freezing at −80°C. Double‐target hybridization experiments were carried out as described in Schmidt et al. (1994). The hybridization solution, containing 2 ng/µL of each probe, 50% (v/v) formamide, 10% (w/v) dextran sulfate, 0.1% (w/v) sodium dodecyl sulfate, and 300 ng/µL sheared salmon sperm DNA in 2× saline‐sodium citrate (SSC), was incubated for 10 min at 70°C and chilled on ice. The hybridization mixture and the chromosomes were denatured together at 70°C in a modified thermal cycler for 8 min, and the temperature was gradually decreased to 37°C. Hybridization was then carried out in a humid chamber overnight at this temperature. Post‐hybridization washes for 10 min each were carried out at high‐stringency conditions (two washes in 2× SSC at room temperature, 20% formamide in 0.1× SSC (v/v) at 42°C, followed by two washes in 2× SSC at 42°C, and two washes in 2× SSC at room temperature). The digoxigenin and biotin at hybridization sites were detected by using sheep anti‐digoxigenin‐fluorescein (Roche) and streptavidin‐Cy‐3 (Sigma), respectively. After counterstaining with DAPI (4′,6‐diamidino‐2‐phenylindole; 0.2 µg/mL 4,6‐diamidino‐2‐phenylindole, in McIlvaine buffer pH 7.0), the slides were mounted in AF1 antifade solution (Citifluor) and examined with a Leica DMRB fluorescence microscope. At least 10 metaphase plates were analyzed, and images were captured using an ILCE‐7 camera (SONY) and optimized using Adobe Photoshop 5.0.

### 2.2 Analysis of TEs in two reference olive genome assemblies

#### 2.2.1 Collection and abundance estimation of full‐length TEs

The genome assemblies of *O. e. europaea* var. *europaea* cv. Leccino (Lv et al., 2024; BioProject PRJCA015700) and *O. e. europaea* var. *sylvestris* (Unver et al., 2017; BioProject PRJNA417827) were retrieved from the China National Center for Bioinformation (https://www.cncb.ac.cn/?lang=en) and the National Center for Biotechnology Information (https://www.ncbi.nlm.nih.gov/), respectively. These genomes were scanned for Class I and II TEs using EDTA v1.9.3 (Ou et al., 2019). EDTA was used to search for LTR‐REs employing a combination of LTR_FINDER v1.06 (Xu & Wang, 2007), LTRharvest v1.5.10 (Ellinghaus et al., 2008), and LTR_retriever v2.5 (Ou & Jiang, 2018). Generic Repeat Finder v1.0.2 (Shi & Liang, 2019) and TIR‐Learner v1.18 (Su et al., 2019) were used for the identification of DNA MITE and TIR elements, respectively. On the other hand, HelitronScanner v1.1 (Xiong et al., 2014) was used to search *Helitron* elements. All program parameters were set automatically according to the default pipeline, and only full‐length TEs were retained for further analyses. To classify LTR‐REs at the lineage level, the elements were annotated based on conserved domains using DANTE v1.1.8, accessible on the RepeatExplorer2 Galaxy website. The annotation was performed with default settings, utilizing the REXdb database of TE protein domains and employing a BLOSUM80 scoring matrix. Protein matches were filtered based on their significance. For abundance estimation, the libraries of all full‐length TEs obtained using EDTA were merged and used to mask both genomes using RepeatMasker v4.1.5 (http://www.repeatmasker.org) with the following parameters: ‐no_is, ‐nolow, ‐X.

#### 2.2.2 Insertion time analysis of full‐length LTR‐REs

The insertion time calculation for LTR‐RE lineages in cv. Leccino and var. *sylvestris* genome assemblies was conducted by computing the distributions of pairwise divergence comparisons between the 5′‐ and 3′‐LTRs. The “stretcher” tool in the EMBOSS v6.6.0.0 suite (Rice et al., 2000) was used to calculate pairwise LTR alignments, applying the Kimura two‐parameter model of sequence evolution (Kimura, 1980). Distance matrices were generated using the “distmat” tool within the EMBOSS suite. To estimate the insertion times of LTR‐RE lineages in the two genome assemblies, a mutation rate of 2 × 1.8 × 10^−9^, that is, twofold the rate calculated for synonymous substitutions in gene sequences of *O. europaea* (Barghini et al., 2015), was used. This adjustment reflects the hypothesis that LTR‐REs accumulate mutations at twice the rate of gene sequences (SanMiguel et al., 1998). Peaks in the frequency distribution represent transposition burst events, where lower divergence values suggest recent proliferation (Mascagni, Usai, et al., 2018). The graphical representation was produced using the “ggplot2” R package v3.4.1 (Wickham, 2011).

#### 2.2.3 Analysis of genes lying in the proximity of TEs

We identified TEs located in the proximity of genes by comparing the genome coordinates of protein‐coding genes with those of TEs in cv. Leccino and *sylvestris* reference genome assemblies, within a maximum distance of 5000 bp upstream and downstream of the genes using the BEDTools “intersect” function (Quinlan & Hall, 2010). The Kyoto Encyclopedia of Genes and Genomes (KEGG) enrichment analysis of genes was performed using clusterProfiler v4.0 (Wu et al., 2021) with default parameters.

## 3 RESULTS

### 3.1 Olive repeatome diversity based on short‐read clustering analysis

To investigate the genomic structure in *O. e. europaea*, we analyzed 20 accessions representing both wild and cultivated genotypes (Table 1). Using SSR analyses, we first assigned these individuals to gene pools based on an extended sample of trees. Three *K* values (2, 3, and 5) were supported by this analysis, and the clustering results are presented in the Supporting Information (Table S2). The four oleasters from Morocco and Spain were consistently assigned to the WW gene pool, whatever the *K* value considered. In contrast, five of the seven oleasters sampled in the central and eastern parts of the Mediterranean Basin were assigned to the wild eastern gene pool at *K* = 3 or 5 (WE). The two remaining oleasters and seven cultivars were not clearly assigned to any gene pool and were thus considered as admixed. Only the cultivars Mari from Iran and Picual from Spain were assigned to a cluster, respectively WE and Q1 (at *K* = 3 and 5, Table S2).

**TABLE 1:** The 11 oleaster and nine olive accessions were used for the analyses. Gene pool membership was determined with the structure analysis.

	Accession	Geographic origin	Gene pool		Accession	Geographic origin	Gene pool
**Oleasters**	Al Ascharinah 9	Syria	WE	**Olive cultivars**	Arbequina	Spain	Admixed
	Maskanli 10	Syria	WE		Canino	Italy	Admixed
	Maskanli 21	Syria	WE		Coratina	Italy	Admixed
	Mazhal 9	Syria	WE		Dolce Agogia	Italy	Admixed
	Oludeniz 1	Turkey	WE		Koroneiki	Greece	Admixed
	Urla 6	Turkey	Admixed		Leccino	Italy	Admixed
	Kitries 6	Greece	Admixed		Mari	Iran	WE
	Bni Harchen 1	Morocco	WW		Olivière	France	Admixed
	Dar Chaoui 1	Morocco	WW		Picual	Spain	Q1
	Talambote 2	Morocco	WW				
	Tarifa 1	Spain	WW				

Abbreviations: WE, wild eastern; WW, wild western.

#### 3.1.1 Repetitive component characterization

To identify the different families of repetitive elements in 20 *O. e. europaea* genotypes, several short‐read clustering trials were carried out to maximize the resolution of genotype‐specific repeats. Specifically, comparative clustering analyses were performed by grouping the accessions into wild and cultivated and conducting separate runs with and without satellite filtering. The filtered comparative clustering runs were annotated to identify and classify interspersed repetitive elements, such as TEs. In parallel, non‐filtered clustering results were used to recover TR monomers, which were further analyzed to characterize the satellite DNA families present in the genomes. The annotated repeats (i.e., interspersed element families and TRs) were then integrated to build a comprehensive library of repeat elements representing the repetitive component of both wild and cultivated *O. e. europaea* genomes. This library was used to map Illumina reads of the different accessions and estimate the overall abundance and composition of repetitive sequences (Table 2).

**TABLE 2:** Genome proportion (%) of repetitive elements in 20 *O. e. europaea* genotypes. Values are reported for each accession, together with group means ± standard deviation for oleasters and cultivated olives. Differences between the two groups were assessed using a two‐sample Welch's *t*‐test.

Oleasters											
Repeats	Al Ascharinah 9	Maskanli 10	Maskanli 21	Mazhal 9	Oludeniz 1	Urla 6	Kitries 6	Bni Harchen 1	Dar Chaoui 1	Talambote 2	Tarifa 1
Class I TE	26.27	25.16	25.57	25.24	25.69	26.67	25.49	24.96	24.33	24.48	24.50
Class II TE	8.34	8.89	8.35	8.92	8.41	8.64	8.14	8.34	8.56	8.28	8.53
Satellite DNA	18.16	18.78	18.58	18.34	13.10	16.56	15.03	21.24	22.29	21.21	21.99
rDNA	0.72	0.65	0.63	0.77	0.44	0.64	0.47	0.55	0.50	1.27	0.55
Not classified	1.34	1.45	1.48	1.46	1.46	1.37	1.38	1.37	1.34	1.34	1.36
Total	54.82	54.92	54.61	54.72	49.10	53.88	50.53	56.45	57.02	56.58	56.93

Olive cultivars									
Repeats	Arbequina	Canino	Coratina	Dolce Agogia	Koroneiki	Leccino	Mari	Oliviere	Picual
Class I TE	27.70	27.08	27.05	26.88	27.55	25.60	27.48	27.23	27.91
Class II TE	8.06	7.86	7.76	8.12	8.02	7.04	7.73	8.08	7.90
Satellite DNA	15.03	16.03	15.66	15.79	16.25	22.76	15.45	15.92	14.89
rDNA	0.56	0.93	0.65	0.28	0.55	0.39	0.72	0.58	0.54
Not classified	1.49	1.65	2.31	2.13	1.51	1.11	1.34	1.56	1.52
Total	52.84	53.55	53.43	53.21	53.88	56.91	52.72	53.38	52.76

Statistical comparison			
Repetitive element	Oleasters mean ± SD	Olive cultivars mean ± SD	*p*‐value
Class I TE	25.31 ± 0.74	27.16 ± 0.67	<0.001^***^
Class II TE	8.49 ± 0.25	7.84 ± 0.33	<0.001^***^
Satellite DNA	18.66 ± 2.94	16.42 ± 2.42	0.078ns
rDNA	0.65 ± 0.23	0.58 ± 0.19	0.424ns
Not classified	1.40 ± 0.06	1.62 ± 0.37	0.105ns
Total	54.51 ± 2.57	53.63 ± 1.29	0.339ns

Abbreviation: TE, transposable element.

***p < 0.001; ns, not significant.

On average, the genome fraction occupied by repetitive DNA in the studied accessions was approximately 54%, ranging from 49.10% in Oludeniz 1 (eastern oleaster) to 57.02% in Dar Chaoui 1 (western oleaster) for the oleasters, and from 52.72% in Mari to 56.91% in Leccino for cultivars (Table 2). REs (Class I TEs) represented a substantial portion of the repetitive component in both groups, ranging from 25.60% in Leccino to 27.91% in Picual, both cultivated varieties. DNA transposons (Class II TEs) showed minor differences across accessions, accounting for approximately 7%–9% of the genome in the analyzed samples.

The other major component of genomes was confirmed to be satellite DNA, which constituted a substantial portion of the repetitive fraction and exhibited marked variability, ranging from 13.10% in Oludeniz 1 (eastern oleaster) to 22.29% in Dar Chaoui 1 (western oleaster).

#### 3.1.2 Analysis of interspersed repeats

To gain deeper insight into the structure and diversity of the repetitive landscape of *O. e. europaea*, the analysis of the interspersed repetitive elements was extended to include classification at both the superfamily and lineage levels, reflecting possible lineage‐specific dynamics (Table S4).

In total, eight lineages (plus one group that could not be annotated) were identified among elements belonging to the LTR‐RE superfamily *Copia* (*Ale*, *Angela*, *Bianca*, *Ikeros*, *Ivana*, *SIRE*, *TAR*, and *Tork*), and seven lineages (plus one group that could not be annotated) were identified among LTR‐RE *Gypsy* elements, including *Chromovirus* (*CRM*, *Galadriel, Reina*, and *Tekay*) and non‐*Chromovirus* lineages (*Athila, Ogre*, and *Retand*). The LTR‐RE *Gypsy/Copia* ratio, reflecting the relative contribution of the two superfamilies, remains relatively stable, ranging from 1.24 in cv. Dolce Agogia to 1.45 in the western oleasters Bni Harchen 1 and Tarifa 1 (Table S4). Among LTR‐REs of the *Copia* superfamily, *Angela* elements consistently showed the highest genome proportion across all genotypes, with slightly higher values in cultivated olives (e.g., up to 2.93% in cv. Dolce Agogia) compared to oleasters. *TAR* and *Tork* also contributed substantially, displaying elevated abundance levels in cultivated olives such as Picual and Arbequina, accounting for up to 2.71% and 2.68% of the genome, respectively. These two lineages also showed a significantly higher abundance in cultivated than in wild olives. In contrast, *Ivana* and *Ale* lineages exhibited low abundance across all genotypes, despite also showing statistically significant differences between the two olive groups. Within the *Gypsy* superfamily, the non‐*Chromovirus* lineage *Athila* showed the highest abundance with a significantly different variability between oleasters and olives (Figure 1), ranging from 3.38% in Oludeniz 1 (eastern oleaster) to 4.41% in cv. Picual. Among *Chromovirus, Tekay* is the most represented lineage, with a genome proportion ranging from 2.55% in Tarifa 1 (western oleaster) to 2.95% in cultivars Koroneiki and Picual.

**FIGURE 1:**
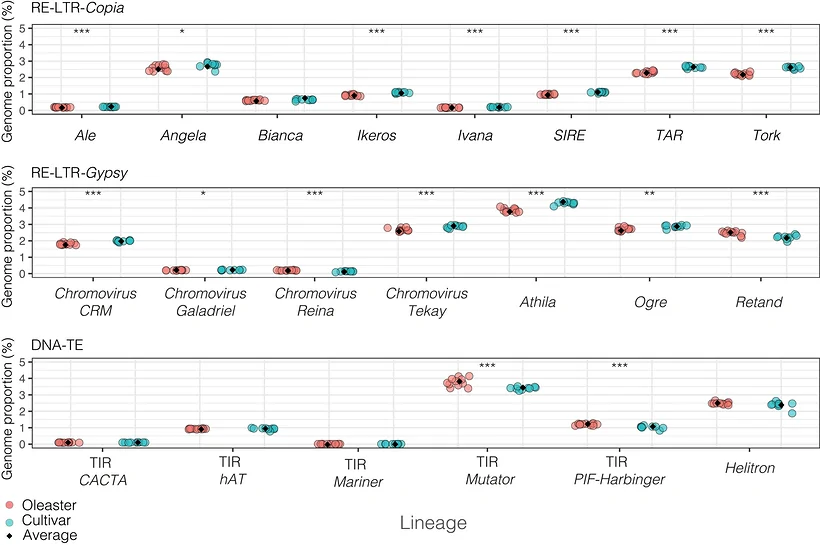
Genome proportion of interspersed repeat lineages in oleasters and cultivated olives. Genome abundance (%) of major TE lineages was compared between oleasters (red) and cultivated olives (blue); each dot represents an individual genotype, and black diamonds represent average values. Black asterisks indicate statistically significant differences between oleasters and olive cultivars. Significance according to Welch's *t*‐test: *p < 0.05, **p < 0.01, ***p < 0.001. LTR, long terminal repeat; RE, retrotransposon.

Regarding Class II TEs, six superfamilies were consistently detected across all analyzed genomes (*hAT*, *Mutator*, *Helitron*, *PIF*‐*Harbinger*, *Mariner*, and *CACTA*). Among these, *Mutator* of the TIR superfamily was the most abundant lineage in both cultivated olives and oleasters, ranging from 3.25% in cv. Dolce Agogia to 4.14% in Mazhal 9 (eastern oleaster), and was significantly more abundant in oleasters than in cultivated olives. *Helitron* was the second most abundant lineage, ranging from 1.87% in Leccino to 2.65% in Oludeniz 1, followed by *PIF‐Harbinger* (0.82% in Leccino to 1.27% in Oludeniz 1), which showed significantly higher abundance in oleasters than in cultivated olives, whereas *Mariner* elements were nearly absent (0.01% in all samples; Table S4; Figure 1).

#### 3.1.3 Analysis of TRs

Clusters of *Olea* sequenced reads classified as putative satellite DNA were manually inspected to validate the monomer consensus sequences of the different TR families. In total, we identified 198 distinct TR sequences, which were grouped by monomer length and sequence similarity into seven major groups of satellite DNA, including five families and three subfamilies within one of them (Figure 2; Table 3). The five families had been previously reported in cultivated olive and brown olives (subspp. *africana* and *cuspidata*) in several independent studies (Barghini et al., 2014; Bitonti et al., 1999; Katsiotis et al., 1998; Lorite et al., 2001; Mascagni et al., 2022; Minelli et al., 2000). Notably, one of these families is shown here for the first time to be organized into three distinct subfamilies (Figure 2), corresponding to Oe‐218, Oe‐80, and Oe‐155, which share part of their monomer sequence but have subsequently diverged into distinct variants (Figure S1).

**FIGURE 2:**
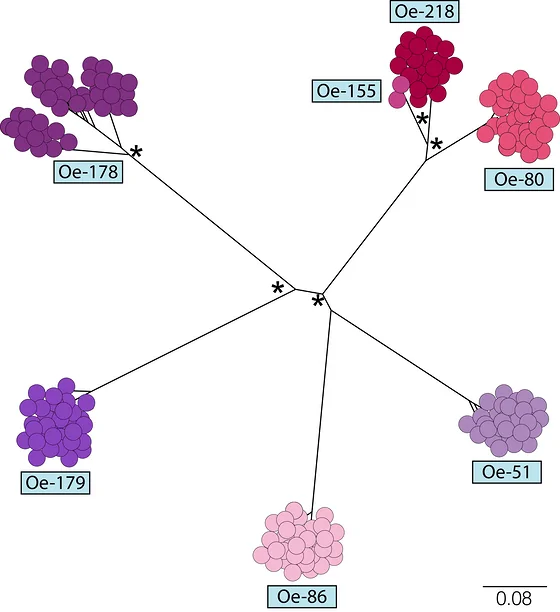
Distance tree of the seven TR families/subfamilies identified across *O. e. europaea* genotypes (198 representative sequences). Bootstrap values higher than 0.6 are shown. Bar length shows the nucleotide distance.

**TABLE 3:** Genome proportion (%) of the seven tandem repeat (TR) families/subfamilies in the 20 *O. e. europaea* genotypes.

Oleasters											
Tandem repeat	Al Ascharinah 9	Maskanli 10	Maskanli 21	Mazhal 9	Oludeniz 1	Urla 6	Kitries 6	Bni Harchen 1	Dar Chaoui 1	Talambote 2	Tarifa 1
**Oe‐51**	0.84	0.68	0.69	0.60	0.58	0.78	0.61	0.53	0.56	0.45	0.50
**Oe‐80**	5.85	7.20	6.52	5.65	3.38	4.73	3.56	7.61	7.66	7.83	8.29
**Oe‐86**	2.41	2.36	2.60	2.77	2.72	2.36	3.48	4.19	4.11	3.87	3.41
**Oe‐155**	0.24	0.35	0.28	0.32	0.18	0.30	0.19	0.25	0.25	0.26	0.26
**Oe‐178**	5.31	4.97	5.50	5.86	3.22	4.78	4.33	6.08	7.09	6.10	6.77
**Oe‐179**	2.86	2.38	2.36	2.32	2.51	2.88	2.34	1.90	1.83	1.94	1.99
**Oe‐218**	0.66	0.83	0.62	0.82	0.50	0.75	0.53	0.66	0.77	0.76	0.76

Olive cultivars									
Tandem repeat	Arbequina	Canino	Coratina	Dolce Agogia	Koroneiki	Leccino	Mari	Olivière	Picual
**Oe‐51**	0.74	0.72	0.70	0.80	0.82	0.68	0.87	0.75	0.78
**Oe‐80**	3.32	3.68	3.57	3.40	3.36	5.48	3.74	3.87	3.56
**Oe‐86**	3.33	3.92	3.63	3.00	4.48	3.07	2.58	3.31	2.89
**Oe‐155**	0.18	0.19	0.18	0.18	0.19	0.28	0.29	0.20	0.18
**Oe‐178**	4.12	4.28	4.41	5.13	4.26	8.26	4.63	4.62	3.84
**Oe‐179**	2.83	2.72	2.63	2.83	2.62	4.32	2.77	2.61	3.13
**Oe‐218**	0.51	0.52	0.54	0.45	0.51	0.68	0.57	0.56	0.52

Oe‐155 was retrieved and further validated through FISH analysis to analyze its genomic distribution compared to that of the closely related Oe‐218. The simultaneous probing of the two TRs confirmed their similarity in chromosomal localization while also supporting the structural identity of Oe‐155 as a distinct satellite subfamily (Figure 3). Oe‐155 hybridization signals of different strength were located on 20 chromosomes (Figure 3B). Most of the signals overlapped with large blocks of DAPI‐positive, pericentromeric or intercalary heterochromatin, whereas only three chromosomes showed weaker signals at one terminal end (Figure 3A,B). Oe‐218 signals were detected on 16 chromosomes (Figure 3C). In this case as well, the hybridization signals were predominantly localized in pericentromeric or intercalary heterochromatin (Figure 3A). Overlap of both signals on the DAPI‐stained chromosomes confirmed that the two sequences colocalized in most regions (Figure 3D). However, only Oe‐155 signals of medium or weak intensity were observed in five chromosomes (arrows in Figure 3B), and only one chromosome bore Oe‐218 signal alone (arrow in Figure 3C).

**FIGURE 3:**
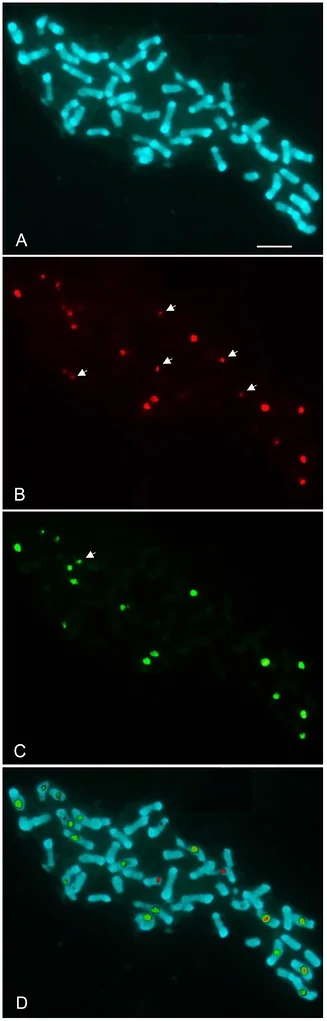
Metaphase plate of *O. europaea* subsp. e*uropaea* cv. Leccino. (A) DAPI staining. (B) Hybridization with probe “Oe‐155” (Cy3). (C) Hybridization with probe “Oe‐218” (fluorescein). (D) Merged image of (B) and (C). Arrows point to chromosomes showing signals related to only one of the two sequences after double‐target probing. Bar = 10 µm.

Analysis of the genomic abundance of TR families across wild and cultivated olives revealed substantial variability in their proportion among genotypes. All TR families were detected in every genome analyzed, but their relative abundance varied considerably. Some families were underrepresented in certain genotypes while showing higher abundance in others, underscoring a high degree of genomic variability in the repetitive landscape of *O. e. europaea* (Table 3). Among oleasters, Oe‐80 exhibited the highest values, with genome proportions ranging from 3.38% in Oludeniz 1 to 8.29% in Tarifa 1, followed by Oe‐178 (3.22%–7.09%) and Oe‐86 (2.36%–4.19%). In contrast, families Oe‐155 and Oe‐218 were consistently less abundant, with values generally below 1%. Among cultivars, Oe‐80 ranged from 3.32% in Arbequina to 5.48% in Leccino, while Oe‐178 ranged from 3.84% to 8.26%, with the maximum rate again peaking in Leccino. Oe‐86 showed considerable variation, with the highest value detected in Koroneiki (4.48%). As in oleasters, Oe‐51, Oe‐155, and Oe‐218 remained the least represented TR families in cultivated genomes (below 1%). Notably, Oe‐51 remained relatively stable across wild and cultivated olives (∼0.5%–0.8%), indicating conserved genomic representation.

Together, these results reveal distinct patterns of TR accumulation between oleasters and cultivated olives. The higher abundance and variability observed in oleasters for some families (e.g., Oe‐80; Figure 4) suggest possible family‐specific dynamics, while localized expansions of some families were observed in some cultivars (e.g., Oe‐178 in Leccino; Table 3). In addition, the combined analysis of sequence variation, genomic abundance, and chromosomal localization supported the characterization of Oe‐155 as a distinct low‐abundance satellite DNA subfamily.

**FIGURE 4:**
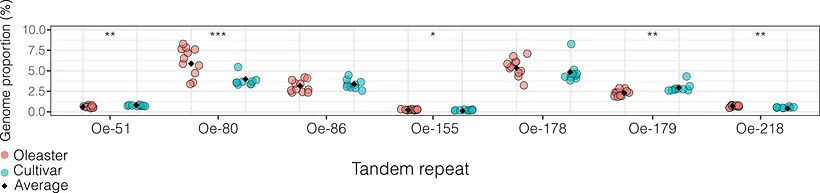
Genome proportion of satellite families and subfamilies in oleasters and cultivated olives. Genome abundance (%) of major TR families/subfamilies was compared between oleasters (red) and cultivated olives (blue); each dot represents an individual genotype, and black diamonds represent average values. Black asterisks indicate statistically significant differences between oleasters and olive cultivars. Significance according to Welch's *t*‐test: *p < 0.05, **p < 0.01, ***p < 0.001.

#### 3.1.4 Comparative analyses of the repeat composition in olives and oleasters

To explore potential variability within lineages and highlight species‐specific repeats, hierarchical clustering was applied to perform a comparative analysis of repetitive DNA content of wild and cultivated *O. europaea* genotypes (Figure 5). Notably, Kitries 6 and Oludeniz 1 (both oleasters from the Aegean area) exhibit a repetitive DNA content profile quite similar to that of cultivated olives. In contrast, Leccino did not cluster with other analyzed accessions, indicating distinct repeat compositions compared to the other cultivated varieties.

**FIGURE 5:**
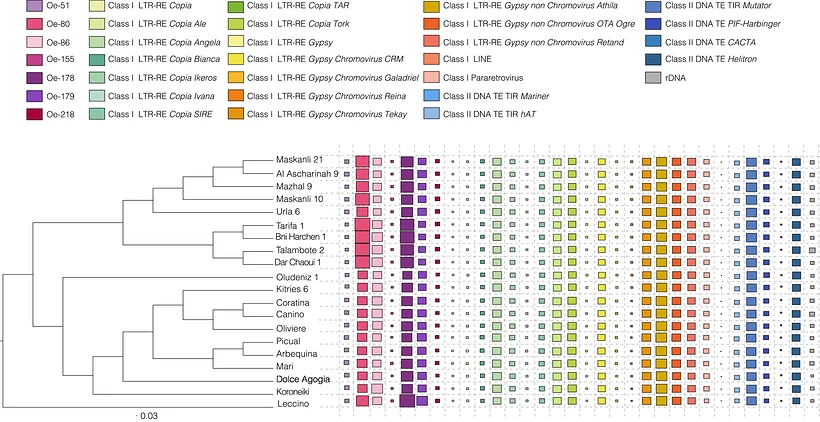
Comparative analysis of repetitive DNA content across wild and cultivated *O. e. europaea* genotypes. The size of the rectangle is proportional to the genome proportion of a cluster for each genotype. The colors of the rectangles correspond to the different repeat families. LTR‐RE, long terminal repeat retrotransposon; TE, transposable element.

To investigate interspecific variability in the repetitive component, a PCA was performed using the genome proportions of all identified repeat families. The analysis revealed a clear separation of genotypes, primarily by habitus (wild vs. cultivated; Figure 6). However, when the analysis was restricted to satellite DNA only (Figure 7A), this separation was no longer observed. In this case, genotypes from both groups were intermixed. Finally, to explore whether the gene pool structure influences the differences observed within the wild olive group, we repeated the satellite‐based PCA considering only the oleaster genotypes (Figure 7B). In this subset, the first principal component primarily reflected geographic origin, with greater variability among eastern oleasters.

**FIGURE 6:**
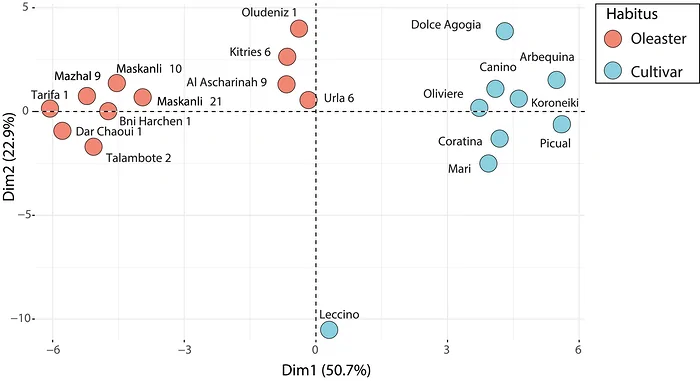
Principal component analysis (PCA) of repetitive DNA content across wild and cultivated *O. e. europaea* genotypes, including both oleasters (red) and cultivated olives (light blue).

**FIGURE 7:**
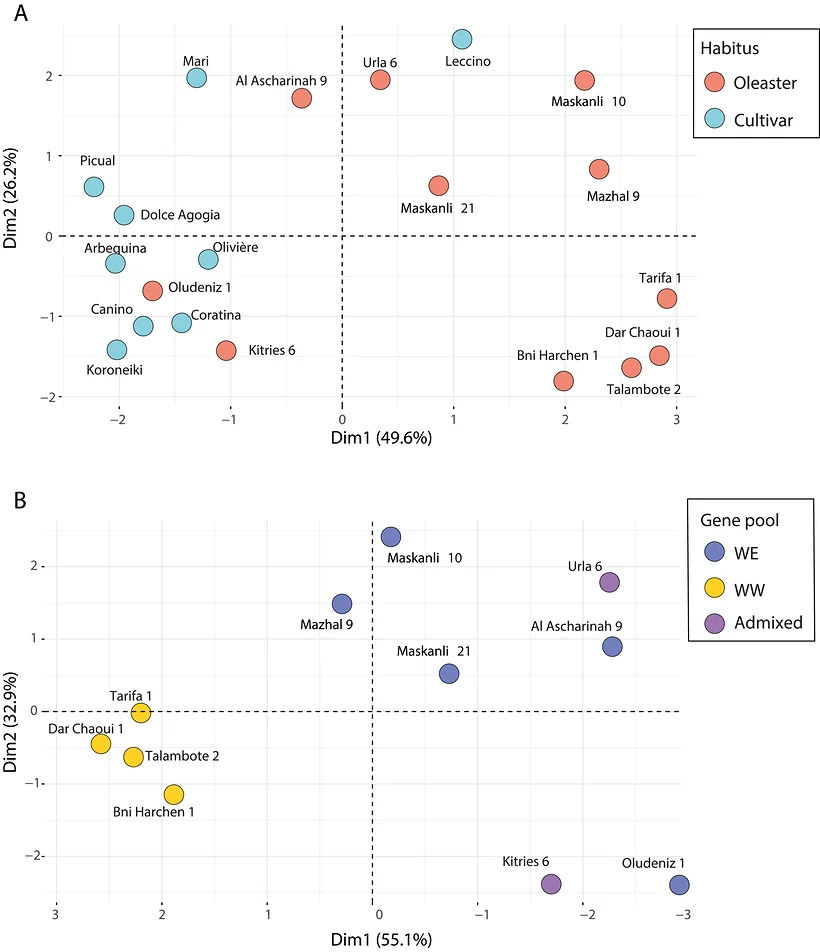
Principal component analysis (PCA) of satellite DNA genome proportions (A) across *O. e. europaea* genotypes, including both oleasters (red) and cultivated olives (light blue), and (B) across wild *O. e. europaea* genotypes (oleasters), highlighting intra‐group variability. Wild genotypes are colored according to the gene pool: wild eastern (WE), wild western (WW), and admixed.

### 3.2 Analysis of reference olive genomes of var*. europaea* and var. *sylvestris*

#### 3.2.1 Collection of full‐length TEs

To complement the broad comparative estimates of repeat abundance obtained from the short‐read analysis of 20 accessions, we investigated the abundance, structural preservation, and dynamics of full‐length TEs in the genome assemblies of two representative genotypes: *O. e. europaea* var. *europaea* cv. Leccino (Lv et al., 2024), and the wild genotype *O. e. europaea* var. *sylvestris* (Unver et al., 2017). The number of full‐length TEs belonging to different lineages of both Class I and Class II is reported in Table 4. The total number of full‐length TEs that could be identified in the two assemblies differed markedly between the two genotypes, with cv. Leccino and var. *sylvestris* showing 20,921 and 8548 elements, respectively.

**TABLE 4:** Number of full‐length transposable elements identified in the reference genomes of *O. e. europaea* cv. Leccino and *O. e. europaea* var. *sylvestris*, specified by transposable element (TE) order, superfamily, and lineage.

Order	Superfamily	Lineage	Leccino	*sylvestris*
Class I	*Copia*	*Ale*	1837	400
		*Angela*	928	68
		*Bianca*	406	36
		*Ikeros*	1260	187
		*Ivana*	751	163
		*SIRE*	334	64
		*TAR*	2280	170
		*Tork*	1925	589
		*ND*	0	21
		Total *Copia*	9721	1698
	*Gypsy*	*Chromovirus/CRM*	1982	286
		*Chromovirus/Galadriel*	322	31
		*Chromovirus/Reina*	195	103
		*Chromovirus/Tekay*	1400	84
		Non*‐chromovirus/OTA/Athila*	1639	168
		Non*‐chromovirus/OTA/Tat/Ogre*	1862	331
		Non*‐chromovirus/OTA/Tat/Retand*	1489	250
		ND	0	55
		Total *Gypsy*	8889	1308
	Unknown		0	267
				
	Total Class I		18,610	3273
Class II	TIR	*hAT (DTA)*	313	393
		*CACTA (DTC)*	308	419
		*PIF/Harbinger (DTH)*	27	137
		*Mutator (DTM)*	623	1233
		*Tc1/Mariner (DTT)*	26	77
	MITE	*hAT (DTA)*	167	364
		*CACTA (DTC)*	73	131
		*PIF/Harbinger (DTH)*	121	531
		*Mutator (DTM)*	432	1659
		*Tc1/Mariner (DTT)*	11	23
	*Helitron*	*Helitron*	210	308
				
	Total Class II		2311	5275
Total TE			20,921	8548

In Class I, the *Copia* superfamily was especially abundant in Leccino, with a total of 9721 full‐length elements. The most represented lineages included *TAR* with 2280 elements, *Tork* with 1925, *Ale* with 1816, and *Ikeros* with 1260. In comparison, *sylvestris* exhibited much lower numbers across all *Copia* lineages, with *Tork* being the most prevalent, accounting for 589 elements. For the *Gypsy* superfamily, Leccino again showed much higher levels, with a total of 8889 full‐length elements compared to just 1308 in *sylvestris*. This pattern was especially evident among non‐*Chromovirus* lineages, with *Ogre* showing 1862 elements in Leccino but only 331 in *sylvestris*. *Athila* accounted for 1639 elements in Leccino and 168 in *sylvestris*, while *Retand* reached 1489 elements in Leccino versus 250 in *sylvestris*. Within *Chromovirus* lineages, *CRM* was the most abundant, with 1982 elements in Leccino and 286 in *sylvestris*.

In contrast to Class I, the trend reversed in Class II transposons; in fact, 5275 full‐length elements were identified in *sylvestris*, more than twice the 2311 recovered in Leccino. This enrichment was consistent across both TIR and MITE subclasses. The genotype *sylvestris* showed significant expansion of *Mutator* elements, with 1233 full‐length copies among TIRs and 1659 among MITEs, compared to 623 and 432 in Leccino, respectively. Similarly, *PIF/Harbinger* elements were more numerous in *sylvestris*, with 137 TIR and 531 MITE elements, compared to only 27 and 121 in Leccino, respectively.

#### 3.2.2 Insertion time of LTR‐REs

The estimation of insertion times for full‐length LTR‐REs in the genomes of var. *europaea* (cv. Leccino) and *O. e. europaea* var. *sylvestris* allowed for the reconstruction of their proliferation dynamics over evolutionary time. Age estimations, based on LTR sequence divergence, revealed that *Copia* and *Gypsy* superfamilies exhibited distinct temporal patterns between the two genotypes (Figure 8). In *sylvestris*, LTR‐REs exhibited a prominent proliferation peak between 15 and 20 million years ago (MYA), indicating a main burst of transpositional activity. By contrast, Leccino displayed a more gradual accumulation of insertions over time, with no sharply defined peak. A small proportion of LTR‐REs (0.91% in *sylvestris* and 0.51% in Leccino) revealed identical LTR sequences, indicating very recent insertion events, possibly occurring after domestication.

**FIGURE 8:**
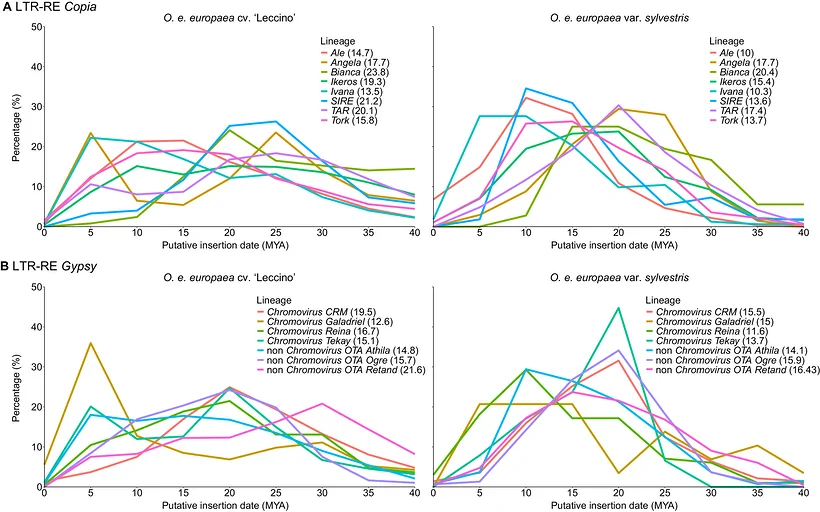
Insertion time of long‐terminal repeat (LTR)‐retrotransposons belonging to (A) *Copia* and (B) *Gypsy* superfamilies in the genome assemblies of *O. e*. *europaea* var. *europaea* cv. Leccino and *O. e. europaea* var. *sylvestris*. For each TE superfamily, the lineages are indicated with different colors. The mean insertion age for each lineage is reported in parentheses. LTR‐RE, long terminal repeat retrotransposon.

Within the *Copia* superfamily, the *Bianca* lineage exhibited the oldest average insertion times in both genotypes, with average values of 23.8 MYA in Leccino and 20.4 MYA in *sylvestris*. In contrast, the youngest proliferation events were associated with the *Ale* and *Ivana* lineages, particularly in *sylvestris*. *Ale* insertions averaged 10.0 MYA in *sylvestris* and 14.7 MYA in Leccino, while *Ivana* insertions averaged 10.3 MYA in *sylvestris* and 13.5 MYA in Leccino. Other *Copia* lineages showed temporal discrepancies between the two genotypes. For instance, *SIRE* elements exhibited a pronounced shift, with an average insertion time of 21.2 MYA in Leccino compared to 13.6 MYA in *sylvestris*. Similarly, *Ikeros* elements appeared older in Leccino than in *sylvestris*, with average insertion times of 19.3 and 15.4 MYA, respectively.

Within the *Gypsy* superfamily, non‐*Chromovirus* elements represented both the youngest and the oldest lineages, with *Athila* averaging 14.8 MYA in Leccino and 14.1 MYA in *sylvestris*, and *Retand* averaging 21.6 MYA in Leccino and 16.4 MYA in *sylvestris*. *Chromovirus* lineages displayed more marked differences. *CRM* elements were significantly older in Leccino, with an average of 19.5 MYA, than in *sylvestris*, averaging 15.5 MYA, and a similar pattern was observed for *Reina*, with averages of 16.7 MYA in Leccino and 11.6 MYA in *sylvestris*. The *Galadriel* lineage displayed peculiar patterns. In Leccino, there was a primary peak around 5 MYA, whereas in *sylvestris*, the main peak was more stretched, between 5 and 15 MYA.

#### 3.2.3 Analysis of gene proximity to full‐length TEs

The genomes of Leccino and *sylvestris* were also analyzed with a focus on TEs located near protein‐coding genes, along with the functional characterization of these latter. In Leccino, 15,086 genes were found in proximity to full‐length TEs, corresponding to 21.5% of the annotated protein‐coding genes, whereas 3316 TE‐proximal genes were identified in *sylvestris*, corresponding to 6.5% (Table S5). The higher number and proportion observed in Leccino may be partly related to the substantially larger number of full‐length TEs recovered from its assembly. After normalization by the total number of full‐length elements, 58.0% of *Copia*, 49.8% of *Gypsy*, and 38.1% of Class II TEs in Leccino were located within 5 kb of a gene, compared with 37.6%, 38.1%, and 28.0%, respectively, in *sylvestris*, indicating a more heterogeneous pattern of gene proximity among TE groups.

The functional characterization of the genes near TEs was narrowed to the top 20 enriched KEGG pathways (Figure 9; Table S5). Most pathways were shared between the two genotypes, including “Ribosome”, which was the most represented among genes near TEs, as well as “Spliceosome”, “MAPK signaling pathway—plant”, “Plant‐pathogen interaction”, “mRNA surveillance pathway”, and “Proteasome”. However, the number of associated genes varied between genotypes. Leccino showed several metabolic and regulatory pathways to be particularly enriched, including “Biosynthesis of cofactors”, “Plant hormone signal transduction”, and “Carbon metabolism”, the latter of which was absent from the top 20 pathways in *sylvestris*. In contrast, *sylvestris* exhibited genotype‐specific pathways that were not among the most enriched pathways in Leccino, including “Cell cycle” and “Ubiquitin mediated proteolysis”. All these observations suggest that the distribution of TEs within the genome is not random and that their retention may be subject to selection dependent on the function of nearby genes. A separate analysis of genes located near *Gypsy* and *Copia* LTR‐REs and DNA TEs showed that “Plant hormone signal transduction”, “MAPK signaling pathway—plant”, and “Plant–pathogen interaction” were significantly enriched among *Copia*‐proximal genes in both Leccino and *sylvestris* (Table S6).

**FIGURE 9:**
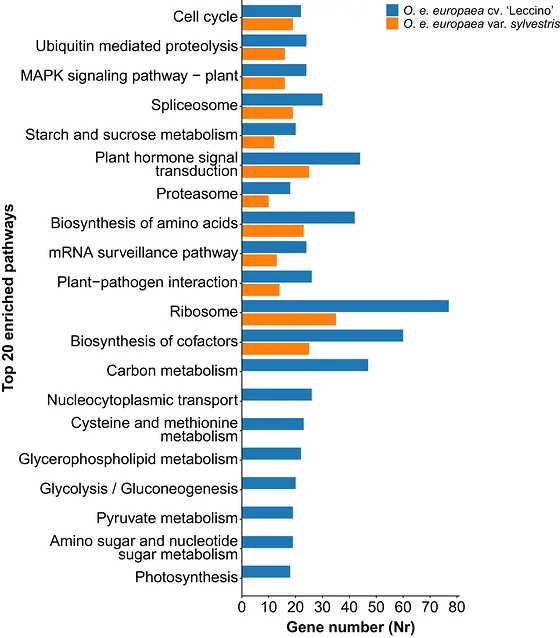
Top 20 enriched Kyoto Encyclopedia of Genes and Genomes (KEGG) pathways among genes located in proximity to transposable elements in the genomes of *O. e. europaea* var. *europaea* cv. Leccino and *O. e. europaea* var. *sylvestris*. Bar length indicates the number of genes associated with each pathway.

## 4 DISCUSSION

Comparative genomics has emerged as a powerful framework for interpreting the evolutionary forces that shape plant genomes, revealing how differences in gene regulation, chromosomal architecture, and repeat dynamics accumulate over time (e.g., Macas et al., 2015; Mascagni et al., 2015, 2022; Wendel et al., 2016). With the increasing availability of high‐quality plant genomes, it has become clear that, alongside recurrent polyploidization events, repetitive sequences are among the most influential drivers of genome diversification, especially in species that share a recent common ancestry but display striking differences in genome size and structure (He et al., 2024). Whereas coding sequences often remain relatively conserved among related taxa, the repetitive fraction shows patterns of much higher variability and is considered a fast‐evolving genomic component (Kuo et al., 2021).

Although the olive genome is known to be particularly rich in repetitive DNA (Mascagni et al., 2022), most previous studies did not capture the extent of repeat intraspecific variation of these sequences. The highly repetitive nature of the olive genome has also been recently supported by the broad repeat annotations of the PacBio HiFi assemblies of Leccino and Frantoio, but that study focused primarily on genome assembly and did not provide a comprehensive characterization of TR families diversity and abundance (Sebastiani et al., 2025). Here, we analyzed the olive repeatome with two approaches describing distinct but complementary dimensions: the short‐read analysis estimates the overall genomic representation of repeat families across multiple accessions, whereas the assembly‐based analysis identifies the intact elements retained within two representative individual genomes.

### 4.1 The olive repeatome (slightly) differs between wild and cultivated trees

Our comparative analyses of repeat composition reveal that the global repeatome retains deep evolutionary signatures separating wild and cultivated olives, consistent with previous genetic and genome‐wide studies (e.g., Besnard et al., 2018; Diez et al., 2015; Gros‐Balthazard et al., 2019; Julca et al., 2020; Zunino et al., 2024). Oleasters display a high abundance and great variability in TRs, as well as significant lineage‐specific differences in several LTR‐RE families, when compared to cultivars. Hierarchical clustering based on the full repeatome generally separates oleasters from cultivars, yet some accessions deviate from this pattern. In particular, Kitries 6 and Oludeniz 1, both collected in the Aegean region, display repeat profiles more similar to those of cultivated olives, consistent with the possibility that they represent feral or admixed individuals shaped by introgression between local oleasters and cultivated germplasm, as documented in several Mediterranean regions (Besnard et al., 2013, 2018; Diez et al., 2015; Khadari et al., 2019). A similar mosaic ancestry can also be observed in Urla 6, known to derive directly from a cross between an ancient cultivar (Safrawi) and a local oleaster. Within the cultivated group, closely related genotypes (e.g., half‐siblings) do not necessarily share a similar repeatome. For instance, Olivière (France) and Picual (Spain) share a parent (Gordal Sevillana as a father; Gómez‐Gálvez et al., 2026; Khadari et al., 2019), and their repeatomes resulted quite similar. In contrast, although the Italian cultivars Leccino and Coratina are also half‐siblings, both descending from Frantoio (Gómez‐Gálvez et al., 2026), their repeatomes are very different. This may also be due to the admixed origin of most cultivars in the central and western Mediterranean, sometimes involving one local wild or feral parent, as was probably the case for Leccino.

Principal component analyses further clarify these patterns. When all repeat families are considered, genotypes segregate by habitus, wild versus cultivated, suggesting that selection during domestication and/or change in their reproductive mode (i.e., sexual vs. vegetative) has promoted diversification of the repeatome between oleasters and cultivated olives. However, this separation collapses when only TR families are considered. When analyzing only TRs, wild and cultivated olive groups are extensively intermixed, supporting the idea that satellite families expand autonomously and evolve rapidly (Garrido‐Ramos, 2015; Plohl et al., 2012; N. Schmidt et al., 2024). When the analysis was restricted to oleasters, it supported a geographic gradient, with Eastern oleasters exhibiting greater variability. This pattern is consistent with longstanding evidence of an East–West diversity gradient in Mediterranean wild olives, reflecting more complex demographic histories and deeper differentiation within the Eastern Mediterranean Basin (Besnard et al., 2013, 2018; Breton et al., 2009; Diez et al., 2015).

### 4.2 Satellite DNA evolution is largely decoupled from olive domestication

The marked expansion of TR families, typical of the *Olea* genus (Barghini et al., 2014; Mascagni et al., 2022), combined with the different abundance of several of them among both cultivated and wild *O. europaea* genotypes, reinforces the view that TRs in the olive tree evolve rapidly and are largely decoupled from the domestication process. This behavior is consistent with what is generally observed for plant DNA satellites, which often undergo rapid, family‐specific expansion and structural diversification (Garrido‐Ramos, 2015), as also broadly reported for satellite DNA evolution across eukaryotes (Biscotti et al., 2015; Plohl et al., 2012). In this context, the emergence of clearly differentiated variants, most notably the newly characterized Oe‐155 subfamily, illustrates how even low‐copy satellites can pursue unique evolutionary trajectories. The difference in the pattern of chromosomal localization of Oe‐155 underscores its structural individuality with respect to the related Oe‐218 and Oe‐80, suggesting that subfamily‐level differentiation is an ongoing process in *O. europaea*, consistent with broader principles of satellite subfamily formation and evolution (Plohl et al., 2012). The sequence and cytogenetic characterization of Oe‐155 therefore constitute a novel outcome of this study, revealing an additional level of satellite DNA diversification and contributing to a better understanding of repeat evolution within *O. europaea*.

### 4.3 TE characterization in two reference olive genomes

After exploring repeatome divergence across multiple wild and cultivated genotypes, we focused on a finer‐scale analysis of TE components by analyzing full‐length TEs in two genomes, for which up‐to‐date genomic resources are available: the recently released high‐quality assembly of *O. e. europaea* var. *europaea* cv. Leccino (Lv et al., 2024) and the reference genome of the wild *O. e*. *europaea* var. *sylvestris* (Unver et al., 2017).

Short‐read‐based and assembly‐based analyses provided complementary views of TE variation in olive. Similar integrative approaches have shown that read‐based estimates of total repeat abundance can be effectively combined with assembly‐based characterization of intact elements to distinguish changes in overall repeat representation from differences in structural preservation and insertion history (Soares et al., 2026; F. Zhang et al., 2023). The different performance of the two approaches was also evident in the analysis of TRs, as the assembly‐based search recovered only a small fraction of the abundance estimated from short reads, consistent with previous observations that repeat collapse can cause mis‐assembly and lead to an underrepresentation of satellite copy number in genome assemblies (Mascagni et al., 2022; Phillippy et al., 2008).

It should also be noted that the two genome assemblies differ in sequencing technology, assembly strategy, and contiguity, which may introduce bias in the quantification of intact TE copies. The lower recovery of intact elements in the *sylvestris* assembly may therefore partly result from fragmentation or collapse of repetitive regions. Consequently, differences in absolute full‐length TE counts cannot be attributed exclusively to biological variation between the two genotypes. However, the full‐length TE analysis was designed to examine structurally resolved elements and their insertion history, rather than to estimate total TE abundance, which was assessed separately through the read‐based analysis.

The Leccino genome carries many more full‐length LTR‐REs than the *sylvestris* one, whereas *sylvestris* harbors more than twice as many full‐length Class II elements. However, repeatome‐based estimates of the genome proportion of Class I and Class II repeats indicate that the relative contribution is broadly similar across many wild and cultivated genotypes. The discrepancy between the two analyses may reflect both methodological and biological factors, as the full‐length TE analysis considers only intact copies and is influenced by assembly quality, whereas genome proportion estimates also include a substantial fraction of fragmented or degenerated elements (Novák et al., 2013; Yu et al., 2024).

Overall, our data suggest that cultivated and wild olives differ mainly in the balance between preservation of complete elements and accumulation of fragmented copies, rather than in their overall TE load. Despite the limitations associated with assembly quality, the contrasting representation of full‐length LTR‐REs and DNA transposons in the two genomes may reflect different patterns of TE retention, turnover, and structural preservation, in line with genome‐wide evidence that TE families can follow distinct evolutionary paths even among closely related genotypes (Quadrana et al., 2016; Tenaillon et al., 2010).

### 4.4 TE proliferation burst mostly predates domestication

Dating LTR‐RE insertions shows that most RE proliferation in olives long predates domestication, with major bursts occurring during the Miocene in the ancestral lineages of both cultivated and wild olives. The more gradual and extended insertion profile observed in Leccino suggests that specific lineages could have continued to accumulate with time. Some LTR‐REs (those exhibiting identical LTR sequences) have inserted very recently, especially in the *sylvestris* genome (0.91% vs. 0.51% in Leccino). The overall difference in the abundance of intact elements between Leccino and *sylvestris* is therefore unlikely to reflect recent RE insertions but rather the long‐term retention or loss of REs inherited from divergent ancestral genetic backgrounds. In this sense, the variability observed today may not originate from differential TE activity per se, but instead from the segregation and fixation of TE‐rich chromosomal regions that were already heterogeneous in the progenitors of both cultivated olive and oleaster. Studies in maize and other species have shown that closely related genotypes can differ substantially in TE content due to the inheritance of distinct haplotypes enriched for TEs (Anderson et al., 2019). Under this scenario, domestication bottlenecks and subsequent vegetative propagation may have fixed particularly TE‐rich haplotypes in cultivated olives (Gaut et al., 2018; Purugganan & Fuller, 2009), whereas wild populations retained a broader mosaic of ancestral chromosomal segments.

The divergent TE landscapes identified between the two genotypes are also reflected in how TEs interact with nearby genes. In olive and in several other fruit tree species, including apple, grapevine, pomegranate, orange, and others, functionally relevant TE insertions in the proximity of genes have been well established (Butelli et al., 2012; Fernandez et al., 2010; Jiménez‐Ruiz et al., 2020; Simoni et al., 2024; L. Zhang et al., 2019). When a TE inserts in the proximity of a gene, it can modify the regulatory sequence controlling gene expression, potentially altering transcription levels (Lisch, 2013). Furthermore, such insertions can influence the expression of nearby genes through epigenetic changes that propagate along the chromosomal locus (Arnaud et al., 2000).

It is interesting to note that Leccino exhibits a much higher number of genes near TEs (15,086 compared to 3316 in *sylvestris*) and that TE insertions occurred in the proximity of genes belonging to over‐represented KEGG pathways, suggesting that TE insertions are at least in part responsible for phenotypic differences between the two genotypes analyzed. For example, in Leccino, the enrichment of pathways related to “Biosynthesis of cofactors” and “Plant hormone signal transduction” suggests that TE insertions may have contributed to shaping some regulatory networks. Conversely, *sylvestris* exhibits enrichment for pathways not prominent in Leccino, including “Circadian rhythm–plant”. This pattern supports the idea that TE insertions near circadian genes enhance their regulatory variability and flexibility for adaptation in wild populations, favoring more stable and predictable rhythmic patterns (Dakhiya et al., 2017; Müller et al., 2016).

### 4.5 Conclusions and future perspectives on the olive repeatome

Overall, our results show that repetitive DNA has been a major and pivotal driver of genome diversification in olive. The variation in satellite families and lineage‐specific LTR‐RE profiles captures both the East/West structure of wild populations and the genomic footprint of domestication. At the genome scale, comparisons between Leccino and *sylvestris* indicate that differences in repeat architecture could largely reflect long‐term turnover and the segregation of TE‐rich chromosomal segments, rather than recent, massive bursts of activity. Finally, the different TE–gene proximity profiles between Leccino and *sylvestris*, together with the consistent enrichment of signaling‐ and stress‐related pathways among *Copia*‐proximal genes in both genotypes, support the view that *Copia* retroelements may preferentially occur near environmentally responsive genes, as previously reported in other plant species (Domínguez et al., 2020; Mascagni, Vangelisti, et al., 2018). These findings suggest that TE distribution may have contributed not only to structural genome divergence but also to differences in the regulatory context of genes involved in environmental responses and phenotypic differentiation.

In our study, we demonstrate that the repeatome differs between wild and cultivated olives, and such variation among gene pools may be useful for investigating the domestication history of the cultivated olive, as done, for instance, for sugarcane (Garsmeur et al., 2025). In particular, due to their abundance, TRs could be relatively easily investigated in degraded DNAs, especially in ancient DNA extracted from archaeological material (Castro et al., 2025; Kistler et al., 2025). At present, however, because of recurrent gene flow between eastern and western populations, it seems difficult to quantify the relative contribution of eastern and western wild gene pools to an ancient genome based solely on TR composition. Additional analyses are still needed to evaluate the discriminatory power of repeat elements for assessing gene pool contributions, especially in ancient samples characterized by limited genomic resolution. Furthermore, the pattern of admixture during the early stages of domestication and subsequent diversification could thus be investigated within a spatiotemporal framework.

Another important question concerns the impact of domestication on the repeatome. Now that we know some genealogical relationships among varieties (Gómez‐Gálvez et al., 2026), it would certainly be relevant to extend this type of analysis to a broader sampling of genotypes by testing the importance of relatedness (comparing full‐ or half‐siblings) and gene pool membership on the repeatome. Farmers have probably selected somatic mutants, and it would be of special interest to investigate the genome of the main cultivated olive progenitors (Diez et al., 2015; Gómez‐Gálvez et al., 2026), which have been propagated for thousands of years in the whole Mediterranean area, and compare them with multiple geographically distinct accessions and wild oleasters. Such an expanded sampling would help clarify how repetitive DNA evolves under prolonged clonal propagation combined with sporadic sexual reproduction events. Centuries of vegetative maintenance may have favored the progressive accumulation of somatic mutations and repeat insertions within clonal lineages. Demonstrating a link between these mutations and agronomic traits of interest in ancient varieties, and subsequently tracking their transmission to their numerous descendants, could provide valuable insights into olive variety breeding.

Future analyses should therefore investigate whether specific classes of repeats tend to accumulate in long‐maintained clones and whether their genomic distribution reflects the demographic and reproductive history of cultivated olives. In particular, insertions occurring within or in proximity to genes associated with agronomically relevant traits could represent an underappreciated source of structural or regulatory variation contributing to varietal diversification.

More broadly, integrating repeatome dynamics with pedigree information, gene pool assignment, and population genomic data may provide new insights into the evolutionary consequences of domestication, introgression with wild populations, and vegetative propagation for the genomic diversification of cultivated olives and help clarify the extent to which repetitive DNA has contributed to shaping olive varietal diversification.

## AUTHOR CONTRIBUTIONS

**Flavia Mascagni**: Conceptualization; data curation; formal analysis; investigation; methodology; supervision; writing—original draft; writing—review and editing. **Samuel Simoni**: Data curation; formal analysis; investigation; methodology; writing—original draft; writing—review and editing. **Gabriele Usai**: Conceptualization; data curation; formal analysis; investigation; methodology; supervision; validation; writing—original draft; writing—review and editing. **Alexis Ryo**: Formal analysis; investigation; methodology; writing—original draft; writing—review and editing. **Marco Castellacci**: Formal analysis; investigation; writing—original draft; writing—review and editing. **Ugo Rogo**: Formal analysis; methodology; writing—original draft; writing—review and editing. **Marilena Ceccarelli**: Formal analysis; investigation; methodology; resources; writing—original draft; writing—review and editing. **Luciana Baldoni**: Resources; writing—review and editing. **Tommaso Giordani**: Formal analysis; investigation; methodology; validation; writing—review and editing. **Lucia Natali**: Data curation; methodology; validation; writing—review and editing. **Andrea Cavallini**: Conceptualization; investigation; methodology; supervision; validation; writing—original draft; writing—review and editing. **Guillaume Besnard**: Conceptualization; data curation; methodology; resources; supervision; writing—original draft; writing—review and editing.

## CONFLICT OF INTEREST STATEMENT

The authors declare no conflicts of interest.

## Supporting information




**Supplementary Figure 1** ‐ Sequence alignments of Oe‐218, Oe‐155, and Oe‐80 TR‐monomer consensus sequences and the FISH probe.

**Supplementary Table S1**. The eleven oleaster and nine olive accessions and the number of Illumina reads used for the analyses.
**Supplementary Table S2‐1**. List of the 241 olive accessions (name, origin) characterized with nuclear microsatellites (SSRs) to assign their genotype to known Mediterranean gene pools. Individual selected for DNA repeats analysis are in bold.
**Supplementary Table S2‐2**. List of loci and primers used for the genetic characterization.
**Supplementary Table S3**. The Oe‐155‐probe, designed on the specific Oe‐155 tandem repeat of *O. e. europaea* and consisting of an arranged array of four monomer repetitions, was synthesized and cloned into the pUC57 vector by Synbio Technologies (New Jersey, USA).
**Supplementary Table S4**. Genome proportion (%) of repetitive elements in 20 O. e. europaea genotypes.
**Supplementary Table S5**. KEGG pathway enrichment analysis of genes nearby TEs in genomes of (A) *O. e. europaea* var. europaea cv. Leccino, and (B) *O. e. europaea* var. *sylvestris*.
**Supplementary Table S6**. KEGG pathway enrichment analysis of genes located in proximity to *Copia*, *Gypsy*, and DNA transposons in the Leccino and *s* *ylvestris* genomes.

## Data Availability

The raw sequencing data have been deposited in the National Center for Biotechnology Information (NCBI) Sequence Read Archive (SRA) database under BioProject PRJNA1445584 and BioSample SAMN02629931 (*O*. *europaea* subsp. *europaea* ‘Leccino’).
