# Supplementary material for: Genome evolution of Mediterranean olives following DNA repeats diversification
Source: Plant Genome. 2026 Oct 15;19(4):e70323. doi: 10.1002/tpg2.70323 (PMC13670204; doi:10.1002/tpg2.70323)
Supplement: Supplementary file 1 [file TPG2-19-e70323-s001.docx]

**Genome evolution of Mediterranean olives following DNA repeats diversification**

Flavia Mascagni^1^*, Samuel Simoni^1^, Gabriele Usai^1^, Alexis Ryo^2#^, Marco Castellacci^1^, Ugo Rogo^1^, Marilena Ceccarelli^3^, Luciana Baldoni^3^, Tommaso Giordani^1^, Lucia Natali^1^, Andrea Cavallini^1^, Guillaume Besnard^2^*

^1^Department of Agriculture, Food and Environment, University of Pisa, Pisa, Italy

^2^Centre de Recherche sur la Biodiversité et l'Environnement (CRBE), Université de Toulouse, CNRS, IRD, Toulouse INP, Université Toulouse 3 – Paul Sabatier (UT3), Toulouse, France

^3^Department of Chemistry, Biology and Biotechnology, University of Perugia, Perugia, Italy

^#^Present address: CEFE, Université de Montpellier, CNRS, EPHE, IRD, Montpellier, France

*Corresponding authors

**Supplementary Figures**

**Supplementary Figure 1** - Sequence alignments of Oe-218, Oe-155, Oe-80 TR-monomer consensus sequences and the FISH probe.
